# The Upregulation of GSTO2 is Associated with Colon Cancer Progression and a Poor Prognosis

**DOI:** 10.1155/2023/4931650

**Published:** 2023-01-11

**Authors:** Xingyu Peng, Jinfeng Zhu, Sicheng Liu, Zitao Liu, Chen Luo, Xun Wu, Zhonglin Yu, Zhengming Zhu

**Affiliations:** ^1^Department of General Surgery, Second Affiliated Hospital of Nanchang University, Nanchang 330006, Jiangxi, China; ^2^Jiangxi Medical College of Nanchang University, Nanchang, China; ^3^Jiangxi Province Key Laboratory of Molecular Medicine, Second Affiliated Hospital of Nanchang University, Nanchang, China; ^4^Department of General Surgery, The Second Xiangya Hospital of Central South University, Changsha, China

## Abstract

Colorectal cancer is the second-leading cause of cancer-related mortality in the United States. Glutathione S-transferase can affect the development of cancer. Glutathione S-transferase omega 2, a member of the GST family, plays an important role in many tumors. However, the role of Glutathione S-transferase omega 2 in the development of colon cancer remains unclear. Herein, our study aimed to investigate the exact role of Glutathione S-transferase omega 2 in colon cancer. We used RNA sequencing data from The Cancer Genome Atlas and the Genotype-Tissue Expression database to analyze Glutathione S-transferase omega 2 expressions. Then, we explore the protein information of Glutathione S-transferase omega 2 in the Human Protein Atlas, GeneCards, and String database. In addition, western blot and immunohistochemistry were performed to evaluate the protein levels of Glutathione S-transferase omega 2 in colon cancer tissues. We acquire data from the Gene Expression Omnibus and The Cancer Genome Atlas databases. Also, we performed relevant prognostic analyses of these data. In addition, we performed a statistical analysis of the clinical data from The Cancer Genome Atlas database and the expression level of Glutathione S-transferase omega 2. Then, we performed Cox regression analysis and found independent risk factors for prognosis in patients with colon cancer. The Kyoto Encyclopedia of Genes and Genomes and Gene Ontology enrichment analyses were used to explore the potential biological functions of Glutathione S-transferase omega 2. The infiltration of colon cancer-immune cells was evaluated by the CIBERSORT method. RNA silencing was performed using siRNA constructs in HCT-116 and HT-29 cell lines. Cell Counting Kit-8 and EdU assays were performed to determine cell proliferation. Transwell experiments and scratch tests were used to determine cell migration. As for the mRNA and protein expression levels of cells, we used quantitative real-time PCR and western blot to detect them. Our research shows that Glutathione S-transferase omega 2 is overexpressed in colon cancer patients, and this overexpression is associated with a poor prognosis. The high expression of Glutathione S-transferase omega 2 is significantly correlated stage with stage, M, and N classification progression in colon cancer by statistical analysis. Univariate and multivariate Cox regression analyses showed that Glutathione S-transferase omega 2 was an independent risk factor for poor prognosis in colon cancer. In addition, we also found that Glutathione S-transferase omega 2 expression levels can affect the immune microenvironment of colon cancer cells. Gene silencing of Glutathione S-transferase omega 2 in HT-29 and HCT-116 cells significantly inhibited tumor growth and migration. In summary, we found that Glutathione S-transferase omega 2 can be used as a molecular indicator of colon cancer prognosis. In vitro, gene silencing of Glutathione S-transferase omega 2 inhibited colon cancer cells' growth and migration.

## 1. Introduction

Colorectal cancer (CRC) is the third most frequently diagnosed malignancy and the third leading cause of cancer-related mortality worldwide [1]. Mortality from colorectal cancer is reduced through screening and early detection [2]. However, most patients are at a late stage when colorectal cancer is diagnosed. Therefore, it is important to identify biomarkers of colorectal cancer cell proliferation to better measure patient outcomes and guide the development of appropriate therapeutic agents.

Glutathione S-transferases (GSTs) are a family of Phase II detoxification enzymes, which are divided into eight distinct classes designated as Alpha, Mu, Pi, Theta, Zeta, Sigma, Omega, and Kappa2 [3–5]. The GST superfamily contributes to many important cellular reactions, including the response to cell proliferation, apoptosis, oncogenesis, tumor progression, and drug resistance [6]. Glutathione S-transferase omega 2 (GSTO2) is a member of the human cytosolic GST superfamily [7]. In recent years, the relationship between GSTO2 and tumor development has received more and more attention. GSTO2 is highly expressed in a wide variety of tumors, for example, urothelial carcinoma, head and neck cancer, bladder cancer, epithelial ovarian cancer, breast cancer, and hepatocellular carcinoma [8–13]. Although the relationship between GSTO2 polymorphism and colorectal cancer susceptibility has been studied [14, 15]. But the mechanism of GSTO2 in colon cancer remains unclear.

This study analyzed the data from The Cancer Genome Atlas (TCGA), Gene Expression Omnibus (GEO), and Genotype-Tissue Expression (GTEx) datasets to evaluate the relationship between the colon cancer patients' clinicopathological characteristics and GSTO2 gene expression with its prognostic significance. In addition, our results showed that gene silencing of GSTO2 significantly inhibited colon cancer cell growth and migration. Finally, due to the importance of the tumor microenvironment in tumor development, we discussed the correlation between GSTO2 expression and colon cancer immune cell invasion.

Our study will help clinicians evaluate the overall prognosis of patients and provide new insights for clinical decision-making and potential therapeutic strategies. However, there were still limitations to our study. The study data were mostly high-throughput gene sequencing data from public databases. Although we verified GSTO2 as a risk factor for colon cancer through in vitro experiments, the direct mechanism of GSTO2's involvement in the occurrence and development of colon cancer remains unclear. So, we will continue to explore the role of GSTO2 in colon cancer in the future.

## 2. Materials and Methods

### 2.1. Pan-Cancer Expression and Prognostic Analysis

The RNA expression and clinical data were downloaded from the TCGA and GTEx databases. Then, the paired and unpaired differences were analyzed and plotted with R. Next, we explored GSTO2's protein expression level in the Human Protein Atlas (HPA: https://www.proteinatlas.org/) database. Also, we used the String (https://string-db.org/) database to build the protein-protein interaction network (PPI) of GSTO2. To visualize the subcellular locations of GSTO2, GeneCards (https://www.genecards.org/) was used to do it. We used R to assess the relationship between overall survival (OS) and GSTO2 expression.

### 2.2. The Data Processing of TCGA and GTEx and GEO

Then, we used Perl to process mRNA data and get 473 colon cancer samples and 41 normal tissue samples. In the same way, 453 colon cancer patients' clinical data were downloaded from TCGA-GDC. Next, the clinical data of 384 cases were obtained after we deleted incomplete and duplicated data. GTEx data were downloaded from the UCEC Xena database (https://xena.ucsc.edu/). In addition, search the keywords “colon cancer survival” and “*Homo sapiens*” in the GEO database (https://www.ncbi.nlm.nih.gov/geo/). Finally, we downloaded the datasets “GSE38832,” “GSE40967,” and “GSE17538.”

### 2.3. Tissue Specimens and Immunohistochemical Staining

30 pairs of colon cancer and para-cancer tissue specimens were obtained from the Second Affiliated Hospital of Nanchang University with the informed consent of the patients. Tissue specimens were fixed with 4% paraformaldehyde and then embedded in paraffin. Slice the tissue into 5 *μ*m slices using a slicer. It was subsequently dewaxed with xylene and water and various concentrations of ethanol.They were then sealed with 10% goat serum after antigen repair. Then, we used anti-GSTO2 (1: 100, Bioss: cat no. bs-16344R) to incubate overnight at 4°C. Following three washes, slides were incubated with secondary antibodies for 30 mins at 25°C. After incubation, DAB was used to stain for 10 min, and hematoxylin was restained for 2 min. Finally, images were taken with a microscope and analyzed with Image-Pro Plus 6.0. This study was approved by the Medical Research Ethics Committee of the Second Affiliated Hospital of Nanchang University.

### 2.4. KEGG and GO Enrichment Analyses of DEGs

The previously obtained data from the GEO database were processed to obtain the differential genes of GSTO2. In addition, we performed GO and KEGG enrichment analyses on the acquisition of differential genes by the R “limma” package. Then, the R “cluster profiler.” “org.Hs.eg.db,” “enrichplot,” and “ggplot2” packages were utilized to perform enrichment analysis on these differentially expressed genes.

### 2.5. Cell Culture and Transfection

Colon cancer HT-29 (catalog numbers: CL-0118), HCT-116 (catalog numbers: CL-0096), SW480 (catalog numbers: CL-0223), and SW620 (catalog numbers: CL-0225B) cells were purchased from Procell (Wuhan, China), and human immortalized colon cells (NCM460, catalog numbers: BNCC339288) were purchased from BNCC (Beijing, China). HT-29 and HCT-116 cell lines were cultured in RPMI‐1640 medium (catalog numbers: 31800, Solarbio, Beijing, China). SW480, SW620, and NCM460 cells were cultured in DMEM culture media (catalog numbers: 12100, Solarbio, Beijing, China). All cell culture media were supplemented with penicillin G (100 *μ*g/mL), streptomycin (100 *μ*g/mL), and 10% fetal bovine serum (FBS; catalog numbers: 164210-50, Wuhan, China), and the cells were grown at 37°C with 5% CO2. The logarithmic growth cells were taken for the experiment. We used Lipofectamine 3000 (Thermo Fisher; catalog numbers: L3000015; Shanghai, China) and GSTO2 siRNA (GenePharma, Shanghai, China; siRNA#1 ID113395, siRNA#2 ID 113396, siRNA#3 ID113397) to make transfections in colon cancer cells based on the provided directions. Western blot (WB) and qRT-PCR were used to detect cell transfection efficiency.

### 2.6. Quantitative Real-Time PCR and Protein Extraction and Western Blot

First, we used the Trizol method to extract total RNA from tissues and cells. Next, we reverse-transcribed it into cDNA (TaKaRa, RR047A) and used it for real-time quantitative PCR (TaKaRa , RR820A). Data analysis was performed using the 2^−ΔΔ^Ct method. The primer sequences used are listed in Table S1. Total protein from HCT-116 and HT-29 cells transfected with or without siGSTO2 was extracted, and western blotting was performed using the following primary antibodies: GSTO2 polyclonal antibody (1 : 500, Proteintech, Wuhan, China, Cat no. 14562-1-AP) and GAPDH monoclonal antibody (1 : 1000, Proteintech, Wuhan, China, Cat no. 60004-1-Ig). HRP-conjugated AffiniPure Goat Anti-Mouse IgG (H + L) (1 : 8000, Proteintech, Wuhan, China, Cat No. SA00001-1); HRP-conjugated AffiniPure Goat Anti-Rabbit IgG (H + L) (1 : 8000, Proteintech, Wuhan, China, Cat No. SA00001-2).

### 2.7. Cell Counting Kit-8 (CCK-8) Assay and 5-Ethynyl-2′-Deoxyuridine Assay (EdU) Assay

The proliferation ability of HT-29 and HCT-116 cells with/without GSTO2 downregulation was observed using the CCK-8 assay and the EdU staining assay. The control and treated cells were seeded in 96-well plates at 5 × 10^3^ cells per group in the CCK-8 assay and incubated until cell attachment occurred. Cell proliferation was detected using a Cell Counting Kit-8 (Bioss, catalog numbers: BA00208). 10 *μ*L of CCK-8 reagent was added to each well at 0 h, 24 h, 48 h, and 72 h after transfection, and the cells were incubated at 37°C for 1.5 h without light. Next, absorbance at 450 nm was measured using a high-performance liquid chromatograph (Agilent, USA), and each experiment was repeated at least three times. As for the EdU assay, we seeded the control and treated cells at 1.5 × 10^4^ cells per group in 96-well plates and incubated them until cell attachment. According to the instructions of the YF®594 Click-iT EdU staining kit (UE, Shanghai, China, catalog numbers: C6017L), the EdU was diluted to 30 *μ*mol/L by the complete medium. Then, we added 100 *μ*L to each well and incubated for 2 h. Next, the medium was removed, and the cells were fixed in 4% paraformaldehyde, neutralized with 2 mg/mL glycine solution, and washed twice with 3% BSA. 0.5% Triton X-100 was used as the osmotic enhancer, and the required Click-iT working solution was configured and incubated for 30 min under dark conditions. 1 × Hoechst 33342 solutions were used for nuclear redyeing. Finally, images were acquired using an Olympus fluorescent microscope (Olympus Corporation, Tokyo, Japan) and analyzed with ImageJ (1.8.0.172).

### 2.8. Wound Healing Assay and Transwell Migration Assay

HCT-116 and HT-29 cells with/without GSTO2 downregulation were digested, centrifuged, resuspended, and counted. Then, the cells were seeded in a 6-well plate at 6 × 10^5^ cells per group. Then, the cells were placed in incubators and incubated. When the monolayer was adherent to the wall, the scratch test was performed with a 200 *μ*L pipetting gun tip. Then, the cells were washed with PBS 3 times, added to a serum-free medium, and placed in an incubator at 37°C. Digital images were acquired using the Olympus CX31 microscope with the DP-26 Olympus digital camera and analyzed by ImageJ (version: 1.8.0.172). In addition, HCT-116 and HT-29 cells with/without GSTO2 downregulation were seeded in transwell chambers at 2 × 10^4^ cells per group. A serum-free medium was used for the upper layer of the chamber, and a complete medium with 15% FBS was used for the lower layer of the chamber. After 48 hours of culture, cells in the lower chamber were fixed with 4% formaldehyde solution and stained with 0.2% crystal violet solution for 20 min. Finally, the cells in the upper layer of the chamber were wiped off with a cotton swab, and the cells that migrated to the lower layer of the chamber were observed and counted by an inverted, phase-contrast microscope (CKK41, Olympus) and analyzed by ImageJ (version: 1.8.0.172).

### 2.9. Statistical Analysis

We performed the Wilcoxon rank-sum test for GSTO2 gene expression differences between tumor and normal tissues, and the Log-rank test was used to analyze survival differences. We used Wilcoxon or Kruskal–Wallis tests and logistic regression to analyze the clinicopathological statistical and GSTO2 gene expression data. Independent risk factors were found by Cox regression analysis, and tumor immune cell infiltration was evaluated by CIBERSORT calculation. R (×64 v.3.5.2), SPSS v23.0, and GraphPad Prism 8 were used for all analyses.

## 3. Results

### 3.1. GSTO2 Expression Analysis in Pan-Cancer

**Figure 1:**
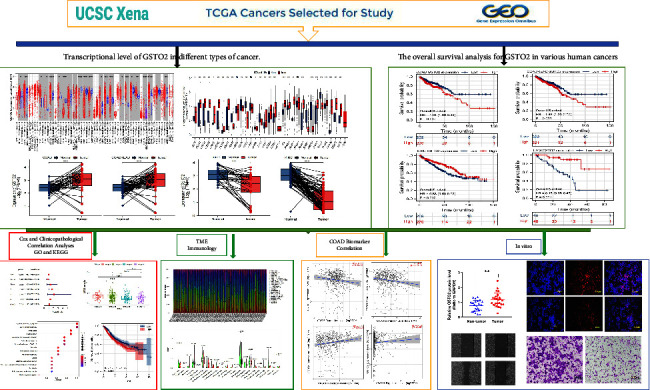
Flow chart of this study.

**Figure 2:**
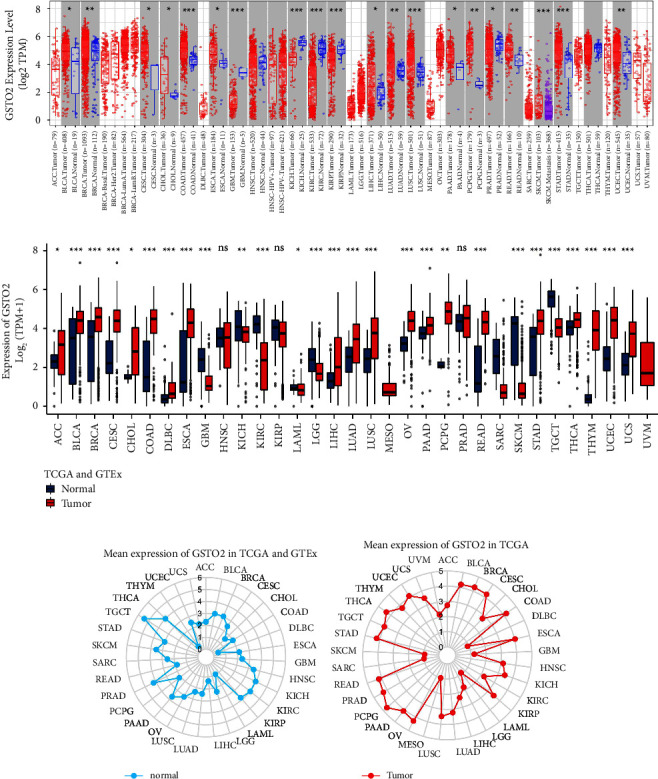
Pan-cancer GSTO2 expression. (a) Pan-cancer expression of GSTO2 in the TCGA database (b) pan-cancer expression of GSTO2 in the TCGA and GTEx databases. (c) GSTO2 expression in normal tissues from the TCGA database. (d) GSTO2 expression in tumor tissues from the TCGA and GTEx databases. The mean value of the GSTO2 expression is represented by dots. ^*∗*^*p* < 0.05; ^*∗∗*^*p* < 0.01 and ^*∗∗∗*^*p* < 0.001; ns, not significant.

The general process of this study is shown in Figure 1. The TIMER2.0 database showed that compared with normal samples, GSTO2was significantly upregulated in 15 cancers (*p* < 0.05), including BLCA, BRCA, CESC, CHOL, COAD, ESCA, LIHC, LUAD, LUSC, PAAD, PCPG, READ, SKCM, STAD, and UCEC (Figure 2(a)). Next, we combined the TCGA and GTEx databases to evaluate GSTO2 expression. The results showed that GSTO2 was highly expressed in 20 tumors: ACC, BLCA, BRCA, DLBC, ESCA, LIHC, LUAD, CESC, CHOL, COAD, LUSC, OV, PAAD, THCA, THYM, UCEC, PCPG, READ, STAD, and UCS (Figure 2(b)). In addition, the normal tissues of humans in GTEx and TCGA. We found the expression of GSTO2 was the lowest in the DLBC, THYM, READ, and COAD para-carcinoma tissues (Figure 2(c)). As for tumor tissues, the highest GSTO2 expression was PCPG, THCA, COAD, STAD, and READ (Figure 2(d)). Furthermore, we plotted a table (Table S2) to show the correlation between GSTO2 mRNA expression levels and various cancer types.

**Figure 3:**
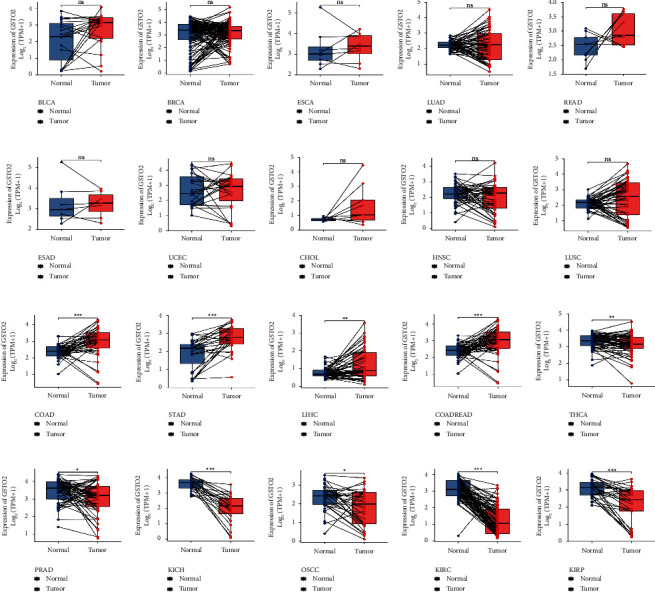
Pan-cancer paired GSTO2 expression. (a–t) Pairings of GSTO2 in multiple tumors were analyzed using information from the TCGA database. Pan-cancer differential expression of GSTO2 in paired tumor and adjacent normal tissues in indicated tumor types from the TCGA database. ^*∗*^*p* < 0.05; ^*∗∗*^*p* < 0.01 and ^*∗∗∗*^*p* < 0.001; ns, not significant.

For paired tumor and normal tissues in TCGA pan-cancer, GSTO2 expression was not significant in 10 cancers (Figures 3(a)–3(j)). Also, GSTO2 was expressed at high levels in COAD, STAD, LIHC, and COADREAD (Figures 3(k)–3(o)), while it was lowly expressed in THCA, PRAD, KICH, OSCC, KIRC, and KIRP (Figures 3(p)–3(t)).

### 3.2. Protein Level of GSTO2

We used the HPA database to explore the protein level of GSTO2 and found that it was lowest in skin cancer and highest in prostate cancer (Supplementary Figure 1A). As for the normal tissues of humans, the high GSTO2 protein expression level in tissues was the fallopian tube, epididymis, prostate, gallbladder, endometrium, and cervix (Supplementary Figure 1B). To explore the subcellular location of GSTO2, we used GeneCards. Then, we found that the GSTO2 protein was most abundant in the cytosol and extracellular (Supplementary Figure 1C). Next, we build the PPI network. Then, we found that GSTO2 was closely associated with IREB2, RBSK, CSS, GRPEL2, NIT1, SELENBP1, GRPEL1, TXN, HINT1, LRRK2, FKBP1A, GPX4, and TFAP2A proteins (Supplementary Figure 1D).

### 3.3. Kaplan–Meier Analysis of Patients from TCGA

**Figure 4:**
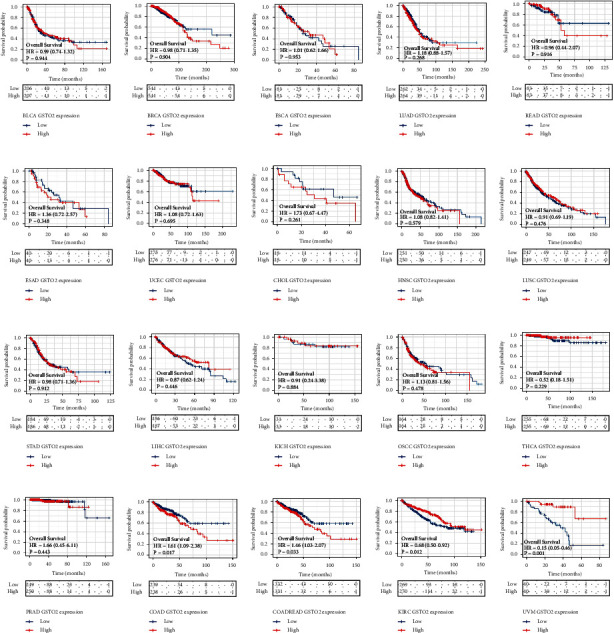
Kaplan–Meier overall survival of GSTO2. (a–t) Pan-cancer Kaplan–Meier overall survival of GSTO2 in indicated tumor types from the TCGA database. The median value of GSTO2 in each tumor was taken as the cut-off value.

For patient data downloaded from the TCGA databases, we used the R “survminer” and “survival” packages to perform a Kaplan–Meier analysis for the overall survival (OS). The results showed that GSTO2 expression differences were not significantly (*p* > 0.05) related to prognosis in 16 cancers (Figures 4(a)–4(p)). Also, GSTO2 was a risk element for patients with COAD and COADREAD (Figures 4(q)-4(r)) and a protective factor for patients with KIRC and UVM (Figures 4(s)-4(t)).

### 3.4. Overexpression of GSTO2 in Colon Cancer

**Figure 5:**
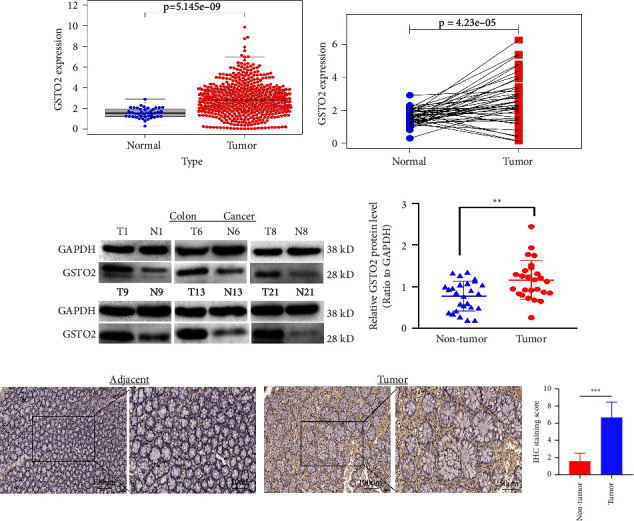
The expression of GSTO2 in colon cancer tissues was significantly higher than that of normal tissues from TCGA. (a) Compared with normal tissues, the expression of GSTO2 mRNA in colon cancer tissues was significantly increased (*p* = 5.145 × 10^−9^). (b) Wilcoxon rank-sum test showed that GSTO2 expression was significantly higher in colon cancer than in adjacent tissues (*p* = 4.23 × 10^−5^). (c–d) Western blot shows that the protein expression levels of GSTO2 in colon cancer tissues and matched nontumor tissues were evaluated (*p* = 0.0016; *n* = 26; T, tumor; NT, nontumor). (e) GSTO2 representative IHC-stained images in colon cancer tissue and adjacent tissues (*n* = 30; magnification: left, 100x; right, 200x). ^*∗∗*^*p* < 0.01 and ^*∗∗∗*^*p* < 0.001.

First, we downloaded GSTO2 mRNA expression from TCGA. Then, we got 473 colon cancer tissue samples and 41 normal tissue samples. We analyzed the difference in GSTO2 expression levels between colon cancer and normal tissues and plotted them in scatter plots. The results (*p* = 5.145 × 10^−9^; Figure 5(a)) showed that the expression level of GSTO2 in colon cancer tissues was much higher than that in normal colon tissues, and statistical analysis of the different results was completed by R. Next, 41 pairs of the 514 samples were analyzed for pairwise differences. A Wilcoxon rank-sum test showed that GSTO2 expression was significantly higher in colon cancer than in adjacent tissues (*p* = 4.23 × 10^−5^; Figure 5(b)). Western blotting was performed after total protein was extracted from tissues. The results showed that GSTO2 protein expression was significantly higher in colon cancer than in adjacent tissues (*p* = 0.0016; Figures 5(c), 5(d)). Similarly, IHC results also showed that the GSTO2 protein was highly expressed in colon cancer (Figure 5(e)). Therefore, we believe that GSTO2 has a high expression level in colon cancer.

### 3.5. GSTO2 High-Expression in Colon Cancer with Poor Survival

**Figure 6:**
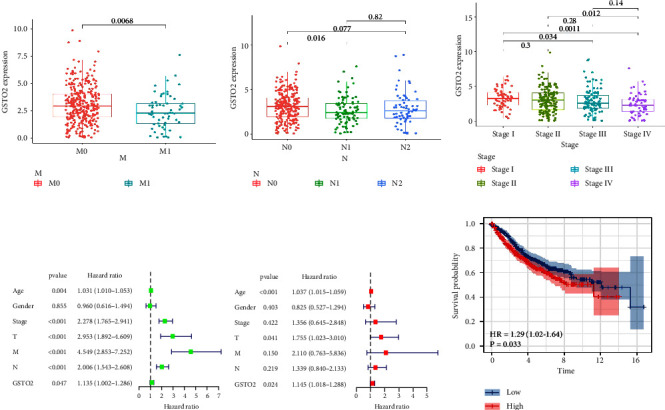
The relationship between GSTO2 expression and clinicopathological characteristics and prognosis. (a–c) Analyze the relationship between GSTO2 expression and N classification, M classification, and stage (Wilcoxon or Kruskal–Wallis test). (d) Univariate Cox regression analysis. (e) Multivariate Cox regression analysis. (f) The overall survival (OS) analysis for GSTO2 in colon cancer patients from the GEO database (*p* = 0.033).

We obtained the information on 453 cases from TCGA and obtained the sample data on 384 cases after removing the duplicate data and incomplete information (Table S3). The Wilcoxon or Kruskal–Wallis test indicated that the overexpression of GSTO2 in colon cancer has a significant correlation with N classification (N2 vs. N1), M classification (M1 vs. N0), and stage (Stage I vs. Stage IV, Stage I vs. Stage III, and Stage II vs. Stage IV) (all *p* < 0.05; Figures 6(a)–6(c)). In addition, we performed logistic regression analysis after grouping based on the median value of GSTO2 expression. The data show that GSTO2 over-expression in colon cancer is related to age (OR = 2.73 for ≤ 65 vs. > 65), stage (OR = 0.37 for Stage IV vs. Stage I; OR = 0.44 for Stage III vs. Stage I; and OR = 0.6 for Stage III vs. Stage II), M classification (OR = 0.55 for M1 vs. M0), and N classification (OR = 0.57 for N2 vs. N0 and OR = 0.50 for N1 vs. N0) (all *p* < 0.05) is significantly correlated (Table S4). In addition, we analyzed the information from 384 cases that we downloaded from TCGA. We performed univariate Cox regression and found that age, stage, T, M, N, and GSTO2 are the factors that affect the prognosis of colon cancer (Figure 6(d)). Also, multivariate Cox regression analysis showed that T, age, and GSTO2 are the factors that affect the prognosis of colon cancer (Figure 6(e)). Next, we downloaded the “GSE38832,” “GSE40967,” and “GSE17538” datasets from the GEO database for external validation of our previous conclusions. After sorting, integrating, and deleting incomplete data, we obtained 831 cases. And Kaplan–Meier analysis was used to assess the relationship between GSTO 2 expression and prognosis in colon cancer patients from the GEO database. The results showed that the high GSTO2 expression group was significantly lower than the low GSTO2 expression group in the survival rate (*p* = 0.033; Figure 6(f)). This was consistent with the results of our previous pan-cancer Kaplan–Meier analysis. The above evidence shows that COAD patients with high GSTO2 expression are more malignant compared to those with low GSTO2 expression.

### 3.6. KEGG and GO Enrichment Analyses of DEGs

**Figure 7:**
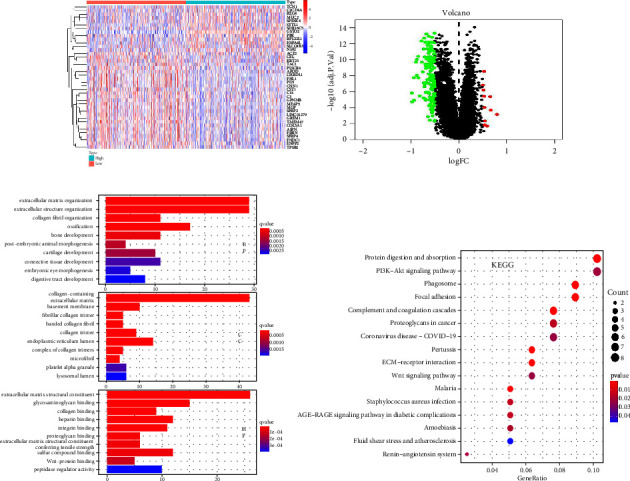
The 168 differential genes are found through the GSE40967, and then these different analysis results are presented by a heat map (a) and a volcano map (b). To explore the potential biological functions of differentially expressed genes, we performed GO (c) and KEGG (d) enrichment analyses.

We selected the dataset “GSE40967” with the most samples to acquire differential genes using the R “limma” package. Next, the samples were grouped according to the median value of GSTO2 expression and filtered according to this standard (logFC > 0.05 and adjusted *p* < 0.05). Then, we got 168 differential genes and the different analysis results were presented by heat map (Figure 7(a)) and volcano map (Figure 7(b)). To explore the potential biological functions of differentially expressed genes, we performed GO and KEGG enrichment analyses. GO enrichment analysis of these genes showed that the main molecular function and cellular composition were “extracellular matrix structural constituent,” “extracellular matrix organization,” “extracellular structure organization,” “endoplasmic reticulum lumen,” “collagen-containing extracellular matrix,” and “glycosaminoglycan binding” (Figure 7(c)). KEGG enrichment indicated that the main enrichment pathways were “Protein digestion and absorption” and “PI3K-Akt signaling pathway” (Figure 7(d)). So, we concluded that the differential genes of GSTO2 may promote colon cancer progression via the PI3K-Akt signaling pathway.

### 3.7. GSTO2 Expression and TME

**Figure 8:**
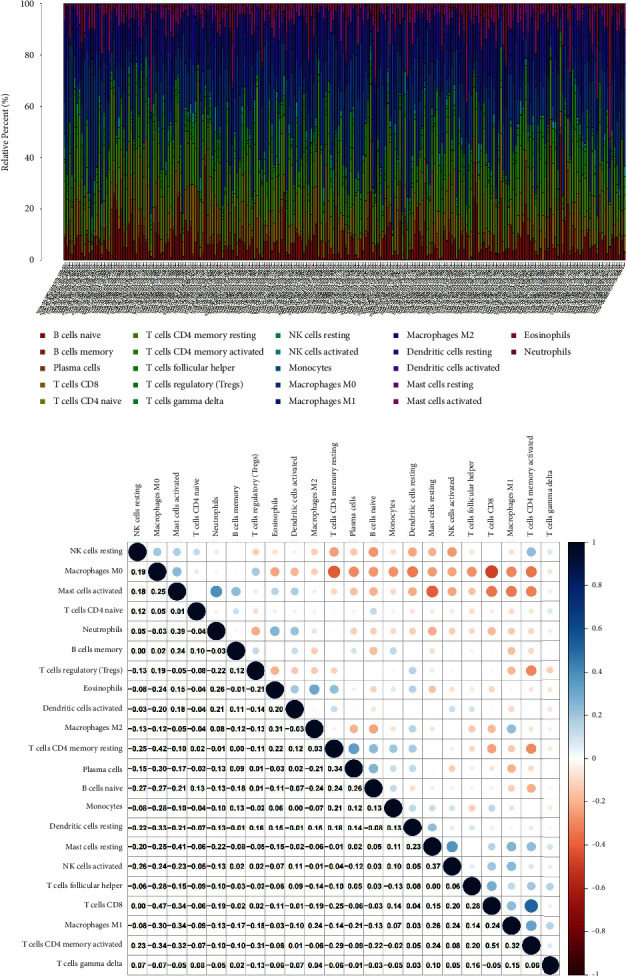
TIC spectrum and correlation analysis in COAD samples. (a) The proportion of 22 TICs in colon cancer was shown in a bar graph. (b) The correlation between 22 TICs was shown with the heat map. The correlation between two immune cells is represented by the corresponding *p* value. The corresponding degree of correlation between the two immune cells is represented by different color depths.

**Figure 9:**
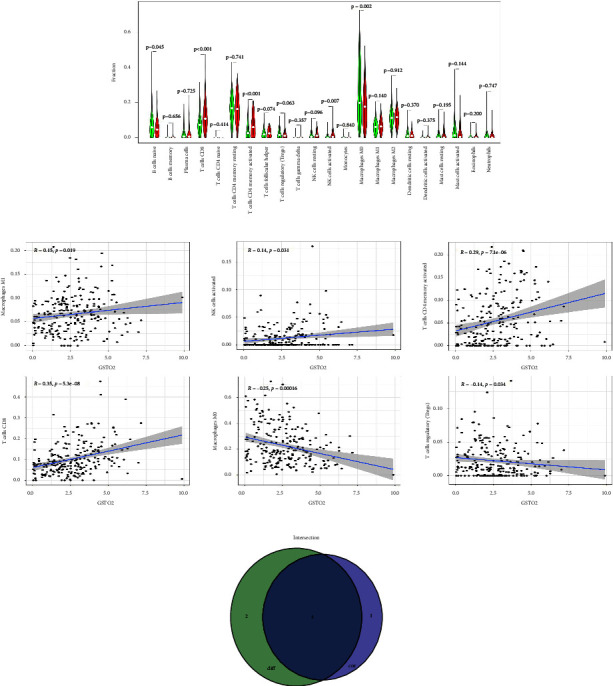
Correlation between GSTO2 expression and TIC ratio. (a) The proportional difference between the GSTO2 high and low expression groups and 22 TICs was shown by the violin chart (Wilcoxon rank-sum test). (b) The correlation between GSTO2 expression and the proportion of TIC was shown by the scatter diagram (Pearson coefficient test). (c) The relationship between different analyses and correlation analyses was shown by the Venn diagram.

We used CIBERSORT to analyze the immunoinfiltration of 22 types of immune cells in colon cancer (Figure 8). There are 5 tumor-infiltrating immune cells (TICs) related to GSTO2 expression, as the analysis results showed (Figure 9(a)). The three positive relationships were T cells CD8, T cells CD4 memory activated, and NK cells activated. The two negative correlations were B cells naive and macrophages. The analysis results of correlation (Figure 9(b)) have shown that there are 6 TICs related to GSTO2 expression (Figure 9(c)). The four positive relationships were NK cells activated, macrophages M1, T cells CD8, and T cells CD4 memory activated. The two negative correlations were macrophages M0 and T cells regulatory (Tregs). The results showed that GSTO2 expression levels can affect the immune microenvironment of colon cancer cells.

**Figure 10:**
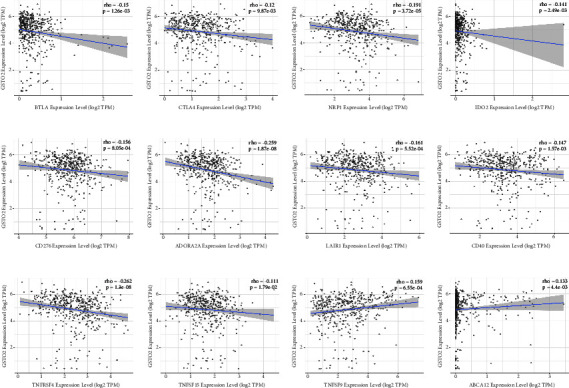
Spearman correlation of GSTO2 expression with BTLA, CTLA4, NRP1, IDO2, CD276, ADORA2A, LAIR1, CD40, TNFRSF4, TNFSF15, TNFSF9, and ABCA12 expression in COAD. (a–j) GSTO2 expression in TIMER2.0 was negatively correlated with BTLA, CTLA4, NRP1, IDO2, CD276, ADORA2A, LAIR1, CD40, TNFRSF4, and TNFSF15 expression in COAD. (k-l) GSTO2 expression in TIMER2.0 was positively correlated with TNFSF9 and ABCA12 expression in COAD.

To analyze the value of GSTO2 in the occurrence and development of COAD, we studied the relationship between GSTO2 and some colon cancer molecular markers through the TIMER database. The results revealed that the expression level of GSTO2 was significantly negatively correlated with BTLA, CTLA4, NRP1, IDO2, CD276, ADORA2A, LAIR1, CD40, TNFRSF4, and TNFSF15 expression in COAD (Figures 10(a)–10(j)), while GSTO2 expression in TIMER2.0 was positively correlated with TNFSF9 and ABCA12 expression in COAD (Figures 10(k)-10(l)).

### 3.8. Determination of GSTO2 Expression in Cell Lines and Transfection Efficiency

**Figure 11:**
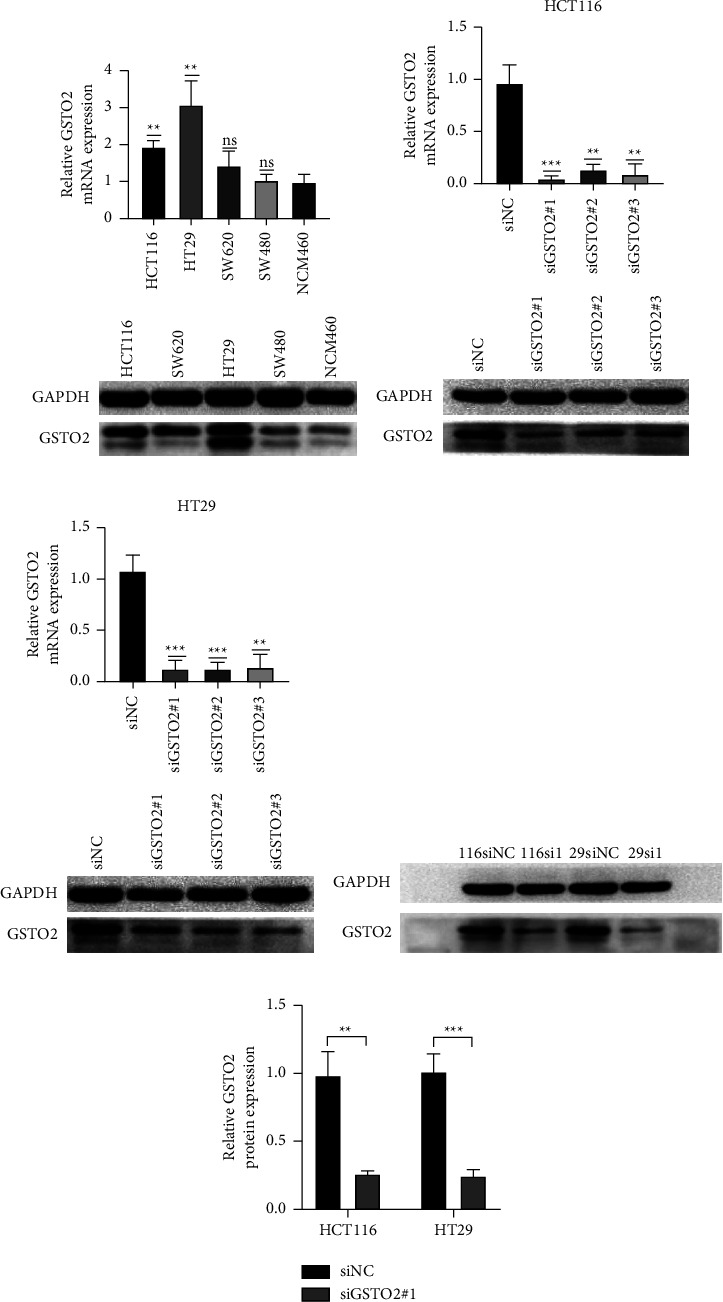
Western blot results show that GSTO2 was expressed higher in HCT-116 and HT-29 cells than in other colon cells (a). Western blot analysis and qRT-PCR corroborate that interference fragments reduced the expression of GSTO2 in HCT-116 (b) and HT-29 cells (c). Western blot analysis (d) and qRT-PCR (e) corroborate that interference fragments siGSTO2-1 reduced the expression of GSTO2 in HCT-116 and HT-29 cells; ^*∗∗*^*p* < 0.01 and ^*∗∗∗*^*p* < 0.001; ns, not significant.

We used western blot to detect the protein expression of GSTO2 in colon cell lines, including HCT-116, SW480, HT-29, SW620, and NCM460. The results indicated that GSTO2 was expressed higher in HCT-116 and HT-29 cells than in other colon cells (Figure 11(a)). Next, HT-29 and HCT-116 were used for follow-up experiments. We used western blot analysis and qRT-PCR to confirm that the interference clips reduced the expression of GSTO2 in HT-29 and HCT-116 cells. The results (Figures 11(b)–11(c)) confirmed that GSTO2 significantly decreased. Subsequently, we used the most effective interference fragment siSTO2-1 to conduct follow-up experiments and verified it again (Figures 11(d)-11(e)).

### 3.9. Lower GSTO2 Expression Restrain the Proliferation and Migration of Colon Cancer

**Figure 12:**
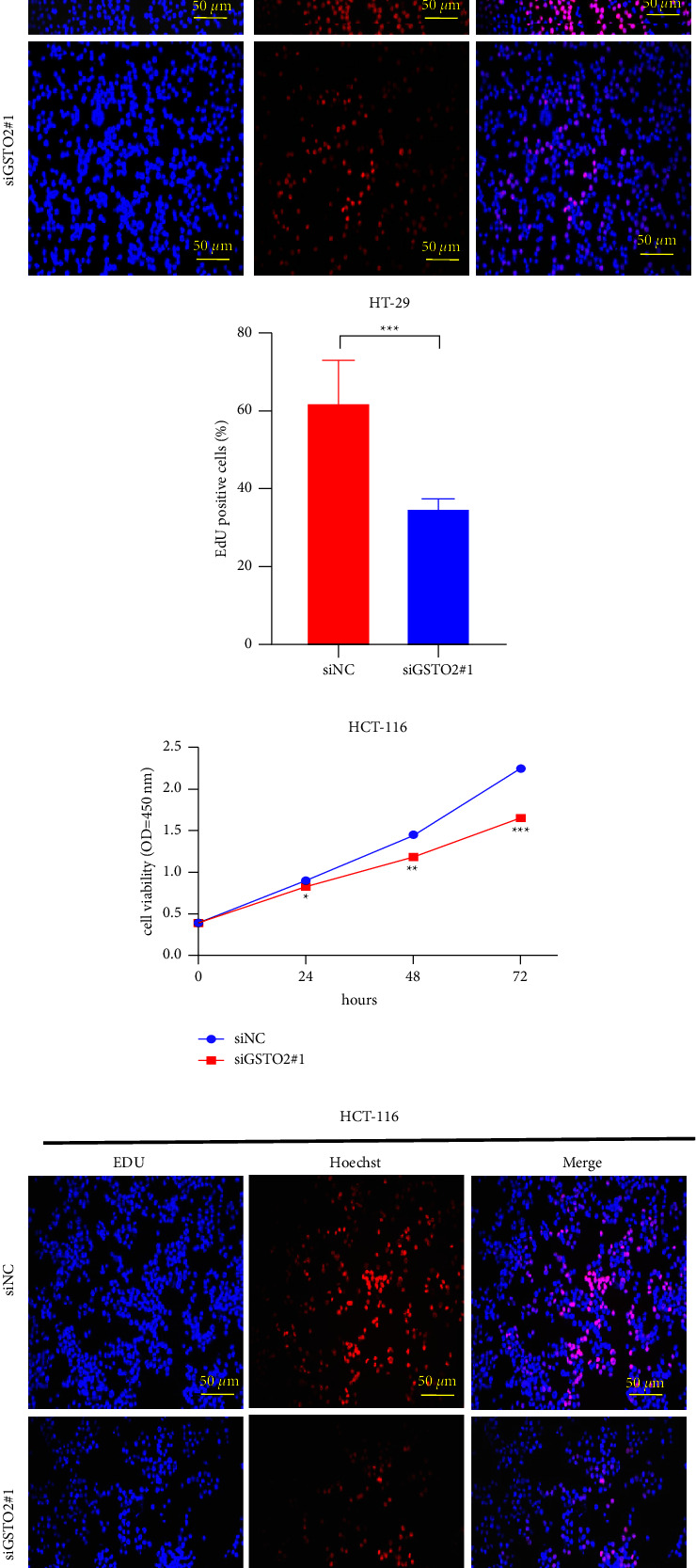
Knockdown of GSTO2 expression inhibits COAD proliferation in vitro. The cell proliferation ability decreased significantly when the HT-29 (a–b) and HCT-116 (c–d) cells were treated with GSTO2 siRNA. Scale bar: EdU, 50 *μ*m ^*∗*^*p* < 0.05, ^*∗∗*^*p* < 0.01, ^*∗∗∗*^*p* < 0.001.

**Figure 13:**
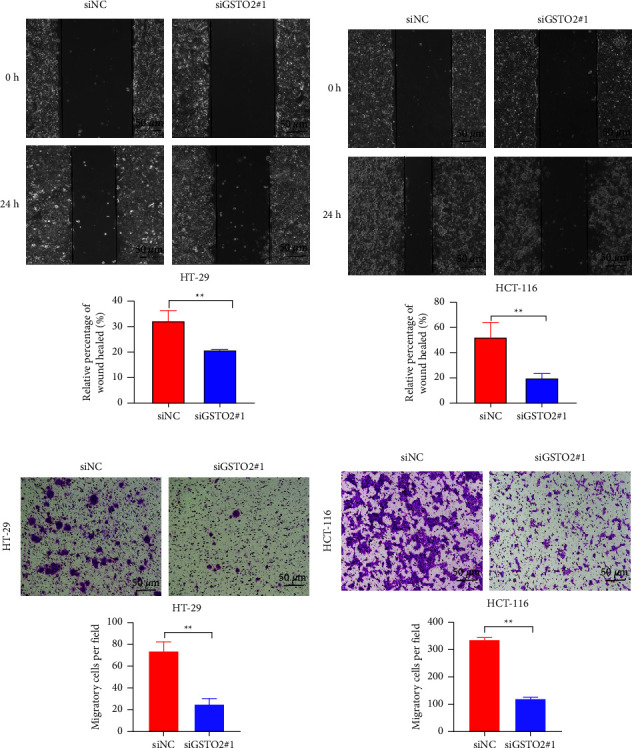
Knockdown of GSTO2 expression inhibits COAD migration in vitro. The wound healing array showed that GSTO2 downregulated HT-29 (a) and HCT-116 (b) cells, which exhibited significantly delayed wound healing compared with controls. Transwell experiments showed that the migratory ability of HT-29 (c) and HCT-116 (d) was inhibited after GSTO2 silencing. Scale bar: transwell experiments and wound healing array, 50 *μ*m. ^*∗∗*^*p* < 0.01.

As the results of the previous clinical correlation analysis indicated, there was a significant positive correlation between the high expression of GSTO2 and the N classification, M classification, and stage of COAD patients. We performed a CCK8 assay and an EdU assay to verify the effect of GSTO2 on the proliferative ability of colon cancer. CCK8 and EdU experiments indicated that cell proliferation was lower in the GSTO2 silenced group than in the siNC group (*p* < 0.05; Figures 12(a)–12(d)). To determine the effect of GSTO2 expression level on the migration ability of colon cancer cells, we performed wound healing and transwell migration experiments. Also, wound healing assays indicated that the rate of migration of HT-29 cells with siGSTO2-1 (Figure 13(a)) was 17.5%, lower than the 45.1% rate of migration of the siNC group. The wound healing assay results (siNC group: 56%, siGSTO2: 10.6%) of HCT-116 cells (Figure 13(b)) were consistent with HT-29 cells. Transwell migration assays indicated that the migration of HT-29 (Figure 13(c)) and HCT-116 (Figure 13(d)) cells infected with GSTO2 siRNA decreased significantly compared with the siNC group.

## 4. Discussion

While CRC morbidity and mortality rates have been declining in North America, Australia, and some countries in Northern Europe, the opposite upward trend has been seen in parts of Asia and South America [16–18]. Large differences in CRC incidence and mortality worldwide are associated with income levels, lifestyle, early diagnosis, and early treatment [19]. More studies on molecular markers related to the pathogenesis and prognosis of colon cancer will help to reduce the morbidity and mortality of colon cancer.

GSTO2 is a member of the human cytosolic Glutathione-S-transferase (GST) superfamily. This superfamily of enzymes can be used to detoxify many conventional chemotherapeutic agents and play an important role in cell proliferation and apoptosis [20]. The role of Glutathione transferase (GST) in redox regulation has been proven to be important for cancer development and progression [21]. According to their properties, they are divided into seven classes: Alpha (A), Mu (M), Omega (O), PI (P), Sigma (S), Theta (T), and Zeta (Z). Many molecules in the GST family play an important role in the treatment and prognosis of tumors. For example, GSTP1 overexpression is a marker of cell proliferation in a variety of tumors, such as transitional cell carcinoma of the bladder [22, 23], renal epithelial renal cell carcinoma [24, 25], ovarian cancer [26, 27], breast cancer [28, 29], and colorectal cancer [30, 31]. GSTM1 and GSTM2 could serve as potential biomarkers of COAD prognosis [32]. GSTO classes include GSTO1 and GSTO2. Under metabolic control, GSTO1-promoted ASC deglutathionylation at the ER is a checkpoint for activating the NLRP3 inflammasome [33]. Some studies show that GSTO1-1 is highly expressed in transitional cell carcinoma [34], esophageal squamous cell carcinoma [35], pancreatic cancer [36], and breast cancer [37]. Xu et al. found that GSTO1 is involved in regulating tumor growth, immune response, and F3 expression, and GSTO1 can be a therapeutic target for cancer [38]. GSTO2 has 70–100 times higher DHA reductase (DHAR) activity than GSTO1 and is considered to be the most active DHAR in mammalian cells [39]. Pongstaporn et al. showed that the GSTO2 polymorphism is associated with ovarian cancer risk [40]. Radic et al. reported that overexpressed GSTO2 in tumor tissue may affect REDOX homeostasis and lead to decreased survival in patients with renal clear cell carcinoma [21]. Qu et al. reported that GSTO2 gene polymorphisms may serve as independent prognostic markers for HCC patients [12]. However, the relationship between GSTO2 and colon cancer has rarely been studied.

We analyzed the difference between the source and the TCGA and GTEx data. Then, as the results show, we found that GSTO2 expression in colon cancer tissues was higher than in normal colon tissues at the mRNA level.Next, we used the HPA database to explore GSTO2's protein level. The result indicated that the protein level of GSTO2 was lowest in skin cancer and highest in prostate cancer. To explore the subcellular location of GSTO2, we used GeneCards. Then, we found that the GSTO2 protein location was mainly in the cytosol and extracellular. Next, we constructed the PPI network. Then we found that GSTO2 was closely associated with IREB2, RBSK, CSS, GRPEL2, NIT1, SELENBP1, GRPEL1, TXN, HINT1, LRRK2, FKBP1A, GPX4, and TFAP2A proteins. Then, the overall survival analysis of those cancers indicated that GSTO2 expression at a high level in colon cancer patients had an association with a poor prognosis. Subsequently, we further explored the relationship between GSTO2 expression levels and colon cancer. So, we think GSTO2 is highly expressed in colon cancer. The Kaplan–Meier OS analysis indicated that GSTO2 is a risk factor for colon cancer patients. In addition, clinical relevance based on the TGCA Database showed that the overexpression of GSTO2 in colon cancer tissues is related to M classification, N classification, and stage, with a poor overall survival rate. This indicates that GSTO2 is highly expressed in colon cancer and is associated with poor survival. Univariate and multivariate Cox regression analyses showed that GSTO2 was an independent risk factor for poor prognosis in colon cancer. These results showed that GSTO2 could be a potential biomarker in colon cancer.

The enrichment analysis of GO and KEGG showed that the high expression of GSTO2 is related to the ECM receptor interaction and may promote colon cancer progression via the PI3K-Akt signaling pathway. As a classical signaling pathway, the PI3K-Akt pathway plays an important role in the occurrence and development of tumors. There have been several studies on colon cancer. For example, Lee et al. pointed out that PI3K/AKT activation induces PTEN ubiquitination and destabilization to accelerate tumorigenesis [41]. Tenbaum et al. showed that *β*-catenin confers resistance to PI3K and AKT inhibitors and subverts FOXO3a to promote metastasis in colon cancer [42]. Research by Khan et al. found that PI3K/AKT signaling is essential for communication in colitis-induced cancer [43].

In recent years, due to the progress of tumor cytology and molecular biology, people have gained a deeper understanding of the relationship between tumors and the environment. The tumor microenvironment (TME) is composed of a variety of immune cells and stromal cells and is critical for tumor initiation and development as well as the regulation of cellular chemotherapy responses [44, 45]. Sanchez-Lopez et al. pointed out that the tumor microenvironment (TME) exerts critical protumorigenic effects through cytokines and growth factors that support cancer cell proliferation, survival, motility, and invasion [46]. Yang et al. demonstrated that CHI3L1, a secretory glycoprotein associated with the TME, could be a target for cancer therapy [47]. TME plays an important role in nanodrugs and immunotherapy [48, 49]. Further research into TME will help improve the efficacy of existing therapies and the development of targeted therapies. Our study found that GSTO2 expression levels can affect the immune microenvironment of colon cancer cells. We used the CIBERSORT algorithm and TIC ratio analysis and found that the expression of GSTO2 has a significant correlation with T cells CD8, T cells CD4 memory activated, NK cells activated, macrophages M1, B cells naive, macrophages, T cells regulatory, and macrophages M0. Therefore, we can speculate that GSTO2 may influence the transition of TME status. In addition, the role of GSTO2 in TME should also be considered when we treat it.

The expression profiles and prognostic values of GSTO2 in COAD were extensively investigated in this in silico work, providing unique insights for future analysis of GSTO2 as prospective targets in COAD. However, our research still has limitations. First, most of the data comes from public databases. To avoid the analysis bias caused by the current retrospective research, we will continue to conduct forward-looking research in the future. Second, while we verified GSTO2 as a risk factor for colon cancer through in vitro experiments, the direct mechanism of GSTO2's involvement in the occurrence and development of colon cancer remains unclear. So, we will continue to explore the role of GSTO2 in colon cancer in the future.

## Data Availability

The datasets presented in this study can be found in online repositories. The names of the repository/repositories and accession numbers can be found in the article/supplementary material.

## Supplementary Materials

Supplementary Materials Supplementary Figure 1: Expression levels of multiple cancer proteins in the HPA database (A). Expression levels of human normal tissue proteins in the HPA database (B). The subcellular location of GSTO2 is listed in the GeneCards database (C). The PPI network for GSTO2's associated proteins (D). Table S1: Primers' target sequences and target sites of siRNA. Table S2: GSTO2 mRNA expression in various human cancers. Table S3: Clinical characteristics of patients with colon cancer. Table S4: GSTO2 expression associated with clinical characteristics (logistic regression). Table S5: Abbreviations.
