# Supplementary material for: The Upregulation of GSTO2 is Associated with Colon Cancer Progression and a Poor Prognosis
Source: J Oncol. 2023 Jan 11;2023:4931650. doi: 10.1155/2023/4931650 (PMC9848813; doi:10.1155/2023/4931650)
Supplement: Supplementary Materials — Supplementary Figure 1: Expression levels of multiple cancer proteins in the HPA database (A). Expression levels of human normal tissue proteins in the HPA database (B). The subcellular location of GSTO2 is listed in the GeneCards database (C). The PPI network for GSTO2's associated proteins (D). Table S1: Primers' target sequences and target sites of siRNA. Table S2: GSTO2 mRNA expression in various human cancers. Table S3: Clinical characteristics of patients with colon cancer. Table S4: GSTO2 expression associated with clinical characteristics (logistic regression). Table S5: Abbreviations. [file 4931650.f1.zip › Table S3 (1).docx]

**Table S3 | Clinical characteristics of patients with colon cancer**

| **Clinical characteristics of patients with colon cancer.** | | |
| --- | --- | --- |
|  | n | Proportion (%) |
| Age |  |  |
| ≤ 65 years | 151 | 39.32 |
| >65 years | 233 | 60.67 |
| Sex |  |  |
| Female | 183 | 47.66 |
| Male | 201 | 52.34 |
| Stage |  |  |
| Ⅰ | 66 | 17.19 |
| Ⅱ | 157 | 40.89 |
| Ⅲ | 100 | 26.04 |
| Ⅳ | 61 | 15.88 |
| T classification |  |  |
| T1 | 8 | 2.08 |
| T2 | 65 | 16.93 |
| T3 | 266 | 69.27 |
| T4 | 45 | 11.72 |
| M classification |  |  |
| M0 | 323 | 84.11 |
| M1 | 61 | 15.89 |
| N classification |  |  |
| N0 | 231 | 60.16 |
| N1 | 87 | 22.65 |
| N2 | 66 | 17.19 |
| Survival status |  |  |
| Death | 78 | 20.31 |
| Survival | 306 | 79.69 |
|  | n | Proportion (%) |
| Age |  |  |
| ≤ 65 years | 151 | 39.32 |
