# Supplementary material for: The Upregulation of GSTO2 is Associated with Colon Cancer Progression and a Poor Prognosis
Source: J Oncol. 2023 Jan 11;2023:4931650. doi: 10.1155/2023/4931650 (PMC9848813; doi:10.1155/2023/4931650)
Supplement: Supplementary Materials — Supplementary Figure 1: Expression levels of multiple cancer proteins in the HPA database (A). Expression levels of human normal tissue proteins in the HPA database (B). The subcellular location of GSTO2 is listed in the GeneCards database (C). The PPI network for GSTO2's associated proteins (D). Table S1: Primers' target sequences and target sites of siRNA. Table S2: GSTO2 mRNA expression in various human cancers. Table S3: Clinical characteristics of patients with colon cancer. Table S4: GSTO2 expression associated with clinical characteristics (logistic regression). Table S5: Abbreviations. [file 4931650.f1.zip › Table S5 (1).docx]

**Table S5 Abbreviations**

|  | Abbreviations |
| --- | --- |
| Glutathione S-transferase | GSTO |
| Glutathione S-transferase omega 2 | GSTO2 |
| Genotype-Tissue Expression | GTEx |
| Human Protein Atlas | HPA |
| Gene Ontology | GO |
| quantitative Real-Time PCR | qRT-PCR |
| The Cancer Genome Atlas | TCGA |
| Gene Expression Omnibus | GEO |
| Kyoto Encyclopedia of Genes and Genomes | KEGG |
| Cell Counting Kit-8 | CCK8 |
| Acute Myeloid Leukemia | ACC |
| Breast invasive carcinoma | BRCA |
| Cholangiocarcinoma | CHOL |
| Diffuse large B-cell lymphoma | DLBC |
| Glioblastoma multiforme | GBM |
| Kidney Chromophobe | KICH |
| Kidney renal papillary cell carcinoma | KIRP |
| Lower Grade Glioma | LGG |
| Lung adenocarcinoma | LUAD |
| Mesothelioma | MESO |
| Pancreatic adenocarcinoma | PAAD |
| Prostate adenocarcinoma | PRAD |
| Sarcoma | SARC |
| Stomach adenocarcinoma | STAD |
| Thymoma | THYM |
| Uterine Carcinosarcoma | UCS |
| UVM | Uveal Melanoma |
| Bladder Urothelial Carcinoma | BLCA |
| Cervical squamous cell carcinoma and endocervical adenocarcinoma | CESC |
| Colon adenocarcinoma | COAD |
| Esophageal carcinoma | ESCA |
| Head and Neck squamous cell carcinoma | HNSC |
| Kidney renal clear cell carcinoma | KIRC |
| Acute Myeloid Leukemia | LAML |
| Liver hepatocellular carcinoma | LIHC |
| Lung squamous cell carcinoma | LUSC |
| Ovarian serous cystadenocarcinoma | OV |
| Pheochromocytoma and Paraganglioma | PCPG |
| Rectum adenocarcinoma | READ |
| Skin Cutaneous Melanoma | SKCM |
| Testicular Germ Cell Tumor | TGCT |
| Thyroid carcinoma | THCA |
| Uterine Corpus Endometrial Carcinoma | UCEC |
