# Supplementary material for: The Upregulation of GSTO2 is Associated with Colon Cancer Progression and a Poor Prognosis
Source: J Oncol. 2023 Jan 11;2023:4931650. doi: 10.1155/2023/4931650 (PMC9848813; doi:10.1155/2023/4931650)
Supplement: Supplementary Materials — Supplementary Figure 1: Expression levels of multiple cancer proteins in the HPA database (A). Expression levels of human normal tissue proteins in the HPA database (B). The subcellular location of GSTO2 is listed in the GeneCards database (C). The PPI network for GSTO2's associated proteins (D). Table S1: Primers' target sequences and target sites of siRNA. Table S2: GSTO2 mRNA expression in various human cancers. Table S3: Clinical characteristics of patients with colon cancer. Table S4: GSTO2 expression associated with clinical characteristics (logistic regression). Table S5: Abbreviations. [file 4931650.f1.zip › WB (1).pdf]

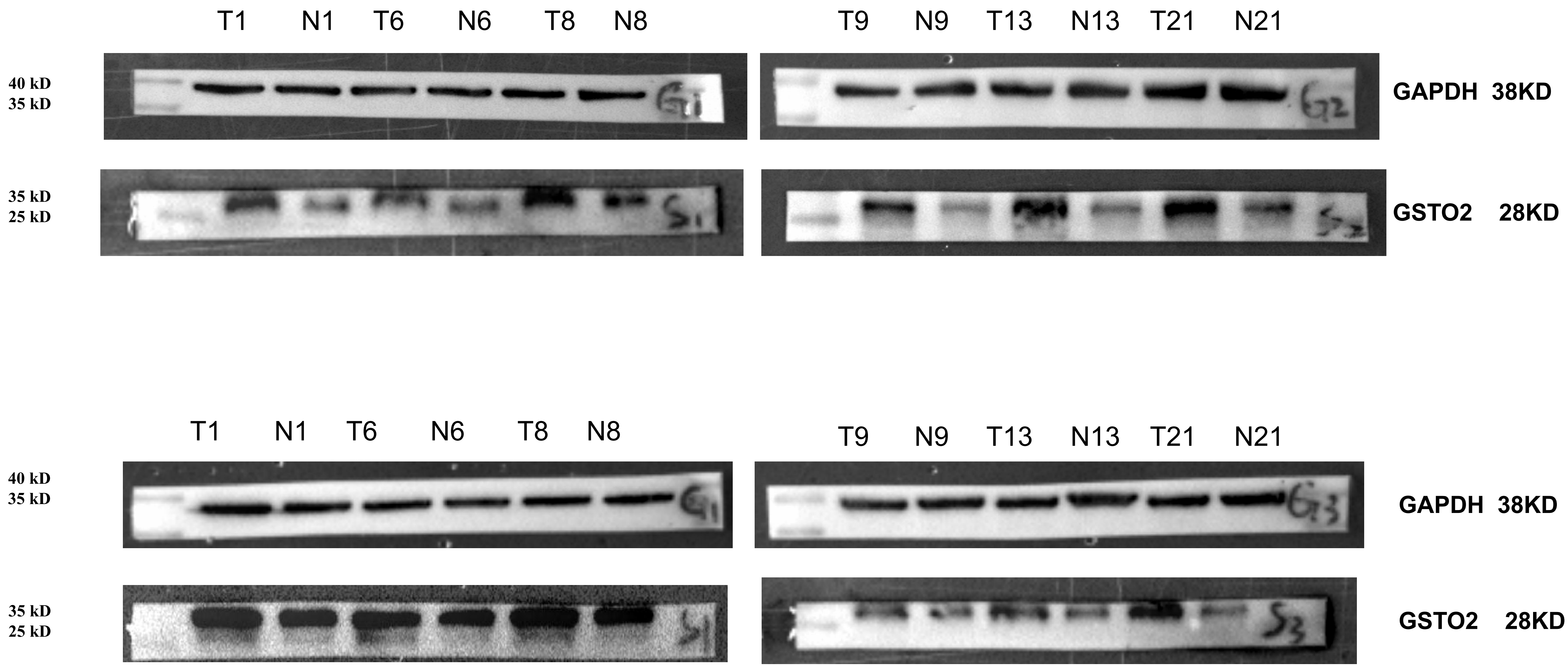
T1 N1 T6 N6 T8 N8
T9 N9 T13 N13 T21 N21
40 kD
35 kD
GAPDH 38KD
35 kD
25 kD
GSTO2 28KD
T1 N1 T6 N6 T8 N8
T9 N9 T13 N13 T21 N21
40 kD
35 kD
GAPDH 38KD
35 kD
25 kD
GSTO2 28KD

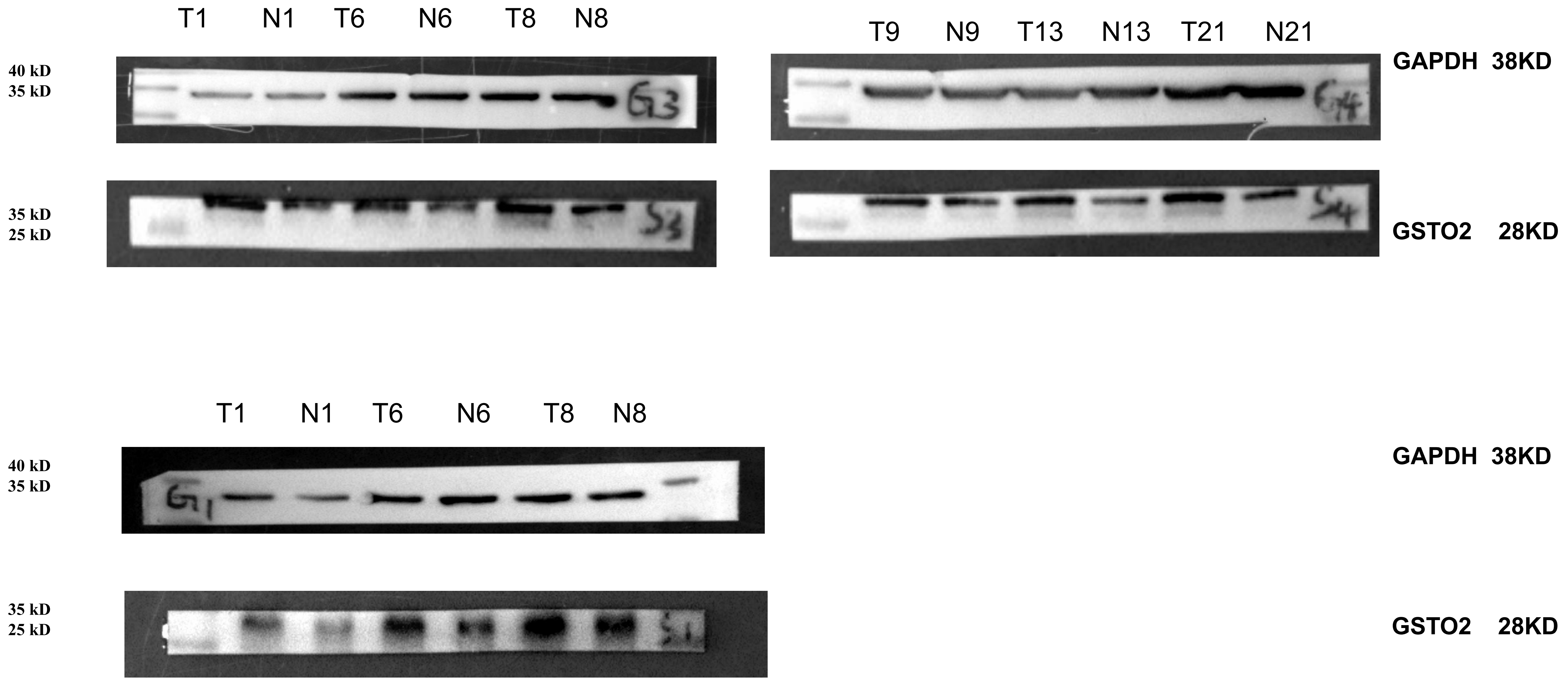
T1
N1
T6
N6
T8
N8
T9
N9
T13
N13
T21
N21
40 kD
35 kD
GAPDH 38KD
35 kD
25 kD
GSTO2 28KD
T1
N1
T6
N6
T8
N8
40 kD
35 kD
GAPDH 38KD
35 kD
25 kD
GSTO2 28KD

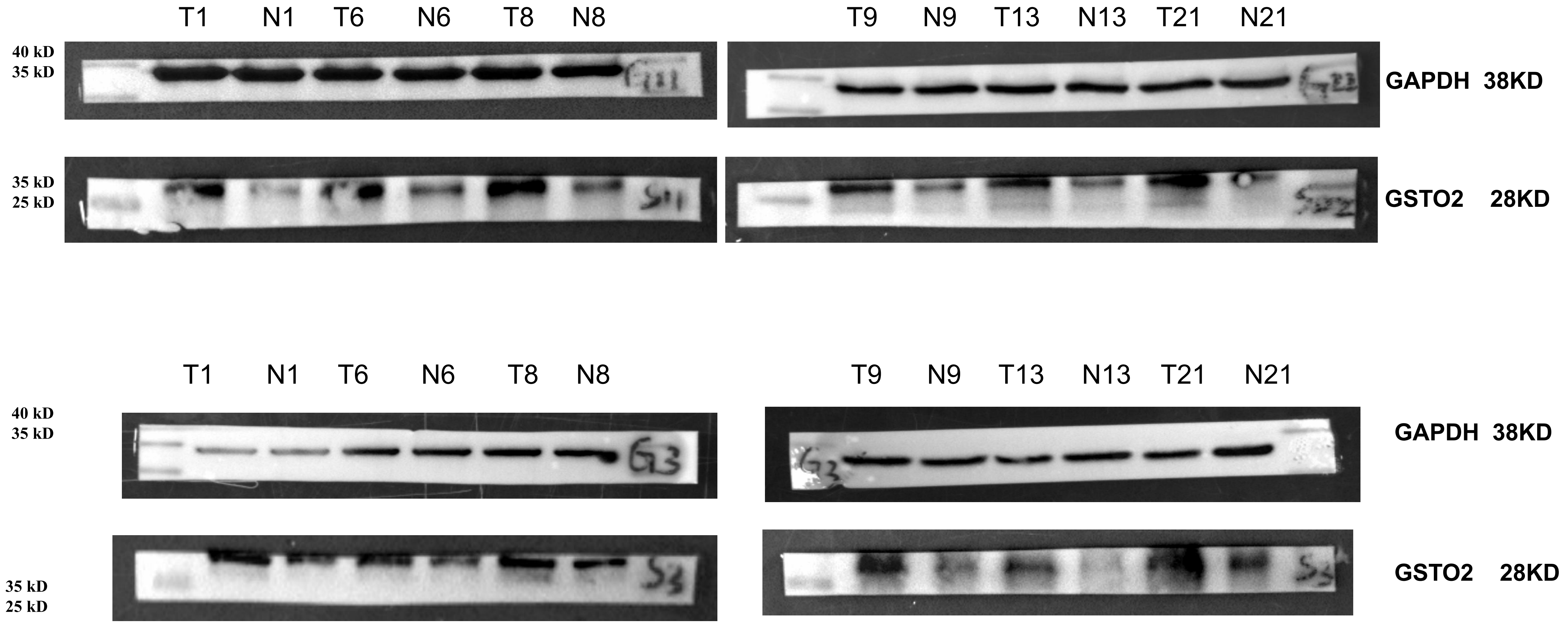
T1 N1 T6 N6 T8 N8
T9 N9 T13 N13 T21 N21
40 kD
35 kD
GAPDH 38KD
35 kD
25 kD
GSTO2 28KD
T1 N1 T6 N6 T8 N8
T9 N9 T13 N13 T21 N21
40 kD
35 kD
GAPDH 38KD
35 kD
25 kD
GSTO2 28KD

Colon Cancer Tissue

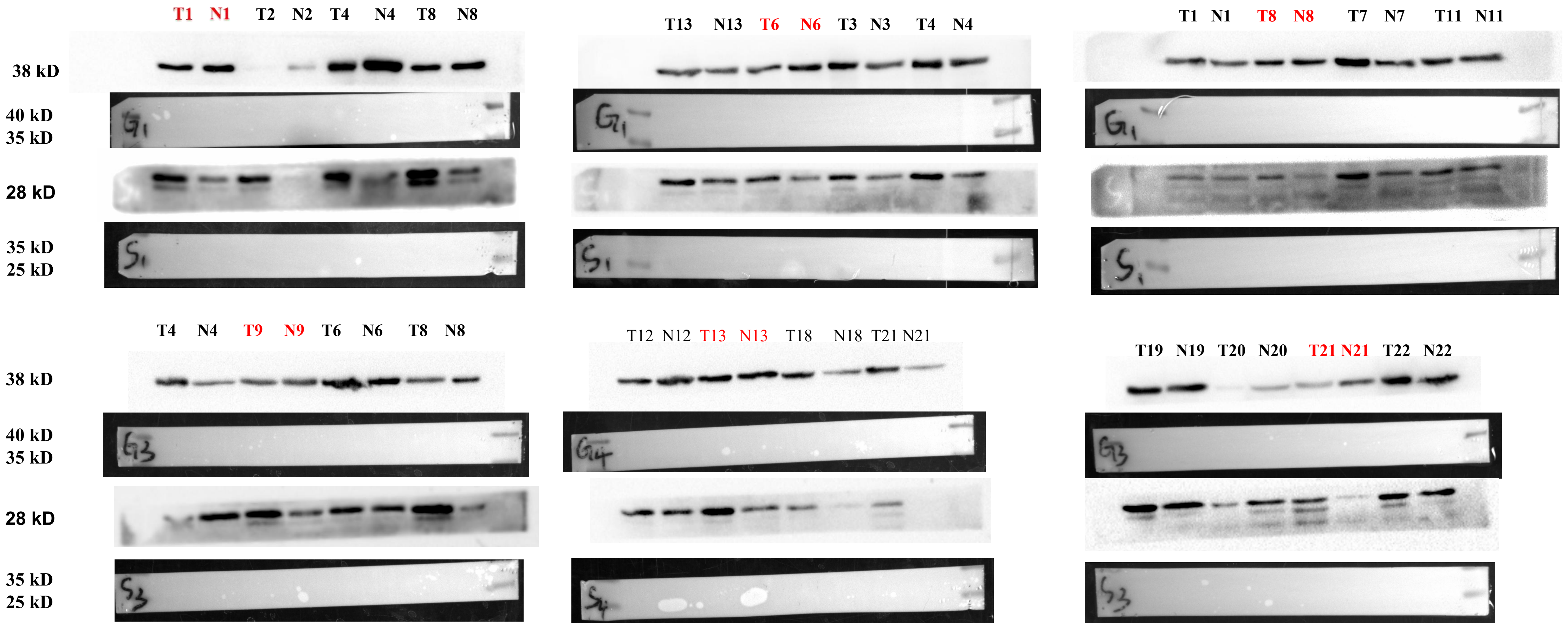

T:Tumor N:Normal The lane marked red is the original image of the tissue WB in Figure 4

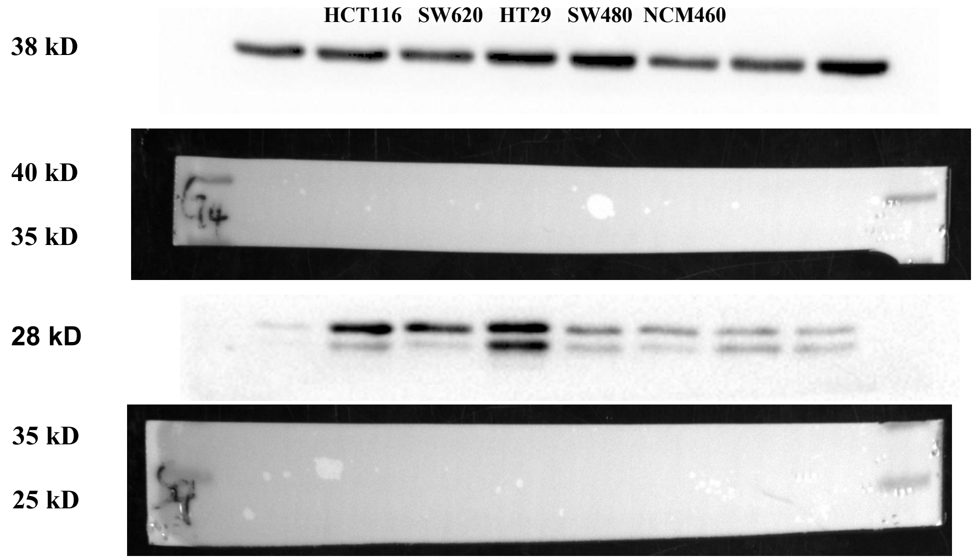

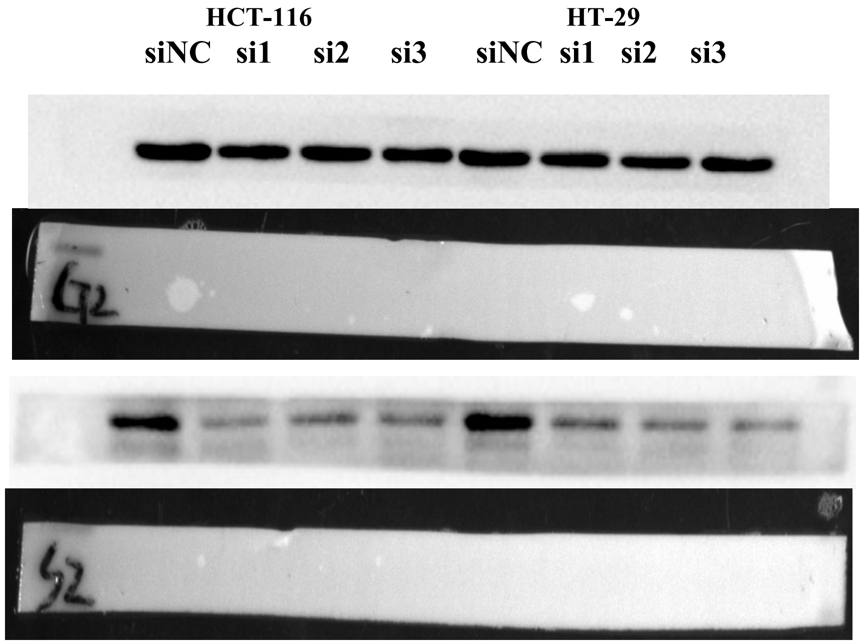

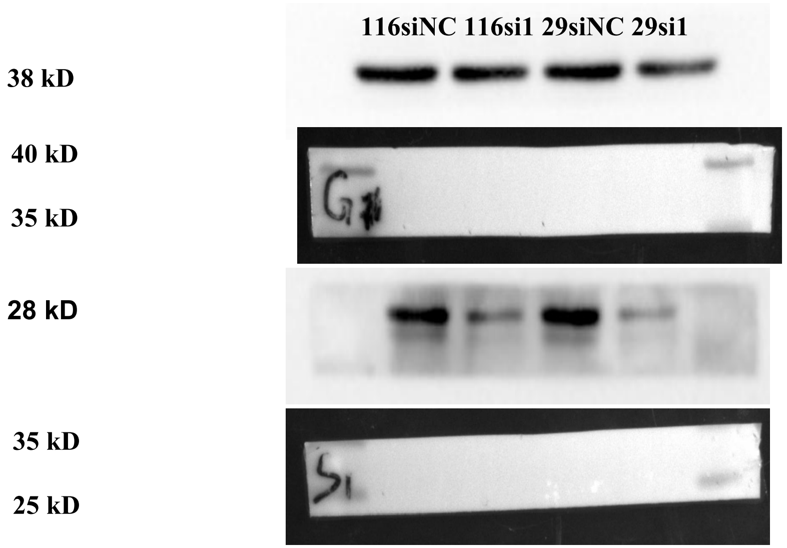

The original file of Figure 11
